# Combining ability analysis of *Cucurbita moschata* D. in Côte d’Ivoire and classification of promising lines based on their *gca* effects

**DOI:** 10.1371/journal.pone.0305798

**Published:** 2024-08-22

**Authors:** Badié Arnaud KOUAGO, Dagou SEKA, Kouakou Fulgence BROU, Beket Severin BONNY, Konan Henri Joel KOFFI, Koffi ADJOUMANI, Raoul Sylvère SIE

**Affiliations:** 1 Université Nangui Abrogoua, Abidjan, Côte d’Ivoire; 2 Ecole Normale Supérieure, Abidjan, Côte d’Ivoire; University of Florida Institute of Food and Agricultural Sciences, UNITED STATES OF AMERICA

## Abstract

*Cucurbita moschata* varieties grown in Africa have very low yield. They have been neglected, and totally ignored in agricultural research programs. However, interest in their fruits, seeds, flowers and leaves is growing nowadays due to their nutritional and medicinal potentials. That growing interest has prompted plant breeders and agronomists to develop research programs for their improvement. A complete diallel cross analysis of four parental lines, *Long*, *Zouan-H*, *Oval*, and *Soubre* and their twelve F1 hybrids, was carried out in a farming environment at the University Nangui Abrogoua, Abidjan, Côte d’Ivoire. The four parental lines and the F1 hybrids were evaluated for their general performances, combining abilities, potency ratio and heterosis effects. The investigated traits included plant height, and eleven fruit- and seed-related characters. The analysis of variance showed significant differences for all traits studied. In addition, the diallel model yielded highly significant *gca* effects of the female parents. The *gca* effects of the male parents were significant for all traits except plant height, length of the fruit, width of the fruit and length of the seed. Highly significant *sca* effects were observed in the crosses for all the traits. Strong maternal effects were observed for the weight and diameter of the fruit, weight of the pulp, number of seeds per fruit, weight of the fresh seeds and 100-seed weight. The general predictive ratio approached the value 1 for all the traits except weight of the fresh seed and width of the dry seed. Most of the characters under this study are predominantly determined by the effects of additive genes. But, weight of the fresh seed and width of the dry seed may be controlled by non-additive genes. Mid-parent heterosis was significant for all measured traits in the crosses, except the length of the fruit. And better-parent heterosis was significant for all traits except plant height, number of fruits per plant and length of the fruit. Gene expression is described by a super-dominance for many traits, and partial dominance for some other traits in all twelve F1 hybrids. Classification of the parental lines based on the effects of their general combining ability grouped the *Soubre* lines as promising contributors to fruit yield. The parental lines *Long* and *Oval* formed another group likely on the basis of the small size of their fruits, the small pulps, the smaller number of fruits per plant and the large number of seeds per fruit. However, *Long* would be a candidate parent for the development of cultivars with longer vegetative growth. The parental line *Zouan-H* formed the third group and it was mostly characterized by its large number of seeds per fruit and relatively large fruits.

## 1. Introduction

*Cucurbita moschata* is an annual crop that is primarily grown for its young shoots, fruits, seeds, flowers and leaves. Its fruits highly contribute balanced nutrition for humans [1]. It is used in various food items. For example, *Cucurbita moschata* powder is used to enhance the β-carotene content of Asian noodles [2]. The flour of the peeled and unpeeled fruit of *Cucurbita moschata* is found comparable to wheat flour [3]. The fruit of *Cucurbita moschata* is often used as an ingredient in baked goods, salami, and sausage [1] for its property as a natural food preservative [4]. It is consumed as pie in North America, and as a stew or a soup in Africa. The unripe fruit is eaten as a boiled vegetable in many regions of the world. The seeds are used with honey to prepare desserts in Central America [5]. In many other countries, the seeds are eaten as snack, after salting and roasting [6]. Almost all organs of *Cucurbita moschata*, except the stem and the roots, are used in human diet. And, for that dietary diversity, *Cucurbita moschata* may be considered as part of the solution to the food challenges associated with the relentless increase of world population. Besides, it may also be considered as one of the most important vegetable crop for humans [7] due to its richness in nutrients and its generous fruit size. *Cucurbita moschata* is relatively high in energy and carbohydrates. It is a good source of vitamins, and its content of carotenoid pigments and minerals are particularly high [8]. It has a very high potential to cover the nutritional needs of the population, particularly vulnerable groups with regard to the need for vitamin A [9]. It is very rich in many essential compounds for the human body. It contains eight amino acids, vitamins A, and C, various minerals, carotene, and trace elements of phosphorus, potassium, calcium, magnesium, and zinc [10]. It contains antioxidants that help to protect the body against free radicals and, to lower the risk of severe diseases. Its nutritional benefits for humans are considerably high. Oil extracted from the seeds of *Cucurbita moschata* is very appreciated due to its high nutritional quality and medicinal properties [5, 6, 11, 12]. The seeds and the fruits of *Cucurbita moschata* have a wide range of bioactive compounds used in the treatment or the prevention of diabetes, cancer, fungal and microbial infections [5, 6, 13]. They are used in traditional medicine in developing countries as well as in advanced countries.

Despite its many nutritional and medicinal benefits, *Cucurbita moschata* remains a marginalized crop. In Côte d’Ivoire, the yield of *Cucurbita moschata* is very insignificant, and the crop has totally been ignored in agricultural research programs. Given all the nutritional potential of this species, consideration should be given to this crop in agronomic research in order to improve its productivity, its fruit and seed yields and its other characteristics of nutritional values to humans such as its leaves. The productivity of this species can be increased by improving the genetic architecture across different cultivars or through the selection of high yielding cultivars [14]. For this, knowledge of certain genetic parameters that govern the important agronomic traits of the crop is necessary, and can be obtained through diallel cross design [15] and implementation. Diallel cross design is used in plant and animal genetic research to estimate general combining ability (*gca*), specific combining ability (*sca*), potency ratio and heterosis for a population from randomly chosen parental lines [16]. Combining ability analysis helps to identify superior parents to be used in breeding programs or to identify promising cross combinations for cultivar development [17]. Crop breeders typically utilize combining ability analysis to choose parents with high general combining ability and hybrids with high specific combining ability effects. General combining ability is a measure of additive gene activity that relates to the average performance of a genotype in a series of hybrid combinations. Specific combining ability evaluates the average performance of certain hybrid combinations compared to the parental lines and is the result of dominance, epistatic deviation, and genotype by environment interactions. Therefore, both *gca* and *sca* effects are important in the selection or development of breeding populations [18]. They are referred to in the selection of parents with the potential to produce hybrids exhibiting greater heterosis. Heterosis is a quantifiable, trait-dependent and environment-specific phenotype, which is the performance of the F1 hybrid exceeding that of the parents [19].

The objectives of this study are to: 1) assess the combining abilities of the parental lines and the progenies from the crosses on the fruit, seed traits and plant height; and 2) to classify parental lines based on their *gca* effects in the *Cucurbita moschata* germplasm development and the improvement of cultivars.

## 2. Materials and methods

### 2.1. Plant material and field experiment

The plant material is composed of the seeds of four inbred lines of *Cucurbita moschata* and their F1 hybrids. They are the parental lines *Oval* (*O*), *Long* (*L*), *Zouan-H* (*Z*), and *Soubre* (*S*), and their F1 hybrids resulting from the complete diallel crosses of these four lines. The seeds of these parental lines come from accessions originating from Korhogo, Ferkessédougou, Zouan-Hounien and Soubre, Côte d’Ivoire, respectively, (Table 1). Accessions of the cultivars *Oval*, *Long*, *Zouan-H* and *Soubre* first underwent four successive cycles of self-fertilization to create the respective inbred lines, *O*, *L*, *Z*, *S*, used in the complete diallel crosses to obtain the seeds of the different families of F1 hybrids. A total of twelve hybrids and the four inbred lines were used in this study.

**Table 1:** Four accessions of *Cucurbita moschata* used in this study and their origins.

Accession	Name	One-letter name	Origin
Korho	*Oval*	*O*	Korhogo (North)
Ferke	*Long*	*L*	Ferkessédougou (North- East)
Zh	*Zouan-H*	*Z*	Zouan-Hounien (West)
Soubre	*Soubre*	*S*	Soubré (South-West)

The experiment was carried out from June to November 2022 on a field of 5673 m^2^ (93 m x 61 m) using a randomized complete block design with three replications, in a farming environment at the University Nangui Abrogoua, Abidjan, Côte d’Ivoire. Each block measured 93 m by 18 m (1674 m^2^), and included 16 plots, randomly made of the twelve hybrids and the four parental lines. Each plot had a surface area of 54 m^2^ (9 m x 6 m) with 3 sowing lines and 4 sowing points per sowing line. Only one F1 hybrid or one parent is randomly assigned to a plot. The experimental field had a total of 576 plants. All the other agronomic management practices, such as weeding, hoeing were applied as recommended, and as needed.

### 2.2. Data collection and analysis

Data were collected at harvest on a sample of 10 plants randomly selected from each of the four parental lines and twelve F1 hybrids in each block. Twelve quantitative characters listed in Table 2 were used to evaluate the hybrids and the parents. The data collected served to compute the descriptive statistics for those characters. An analysis of variance was performed with the measured traits serving as the response variables. Blocks and the 16 genotypic strains were the factors. When significant differences were observed, the method of Tukey was used to separate means due to the genotypic effect of the strains at the 5% level of probability. In addition, the effects of the general combining ability (*gca*) of parent *i* and parent *j* and the specific combining ability (*sca*) of the cross between two parents *i* and *j* were determined according to the following expressions [15], $gca_{i}={\bar{Y}}_{i.}-\bar{Y}..$; $gca_{j}={\bar{Y}}_{.j}-\bar{Y}..$; and $sca_{ij}=Y_{ij}-{\bar{Y}}_{i.}-{\bar{Y}}_{.j}+\bar{Y}..$ where *gca*_*i*_ and *gca*_*j*_ are the *gca* effects of parent *i* and parent *j*, respectively, and *sca*_*ij*_ is the *sca* effect of the cross, ${\bar{Y}}_{i.}$ is the mean of all crosses with the same parent *i*; ${\bar{Y}}_{.j}$ is the mean of all crosses with the same parent *j*; *Y*_*ij*_ is the mean of the crosses between parent *i* and parent *j*; and $\bar{Y}..$ is the mean of all crosses.

**Table 2:** Traits of *Cucurbita moschata* evaluated in this study, along with their descriptions and units of measure in parenthesis.

Abbreviated Name of Traits	Description and (unit of measure)
PLH	Plant height (cm).
NFP	Number of fruits per plant (unit).
LOF	Length of fruit (cm).
LAF	Width of fruit (cm).
DOF	Fruit diameter (cm).
WOF	Weight of fruit (g).
WTM	Weight of the pulp (g).
NOS	Number of seeds per fruit (unit).
WFS	Weight of the fresh seeds per fruit (g).
W100	Weight of 100 dry seeds per fruit (g). Obtained after placing the fresh seeds in an oven for 3 days at 70°C.
LDS	Length of a dry seed (mm). Average of longest axis of 30 seeds
WIS	Width of a dry seed (mm). Average of second longest axis of 30 seeds.

The genetic variances of the *gca* and *sca* effects were assessed with the linear diallel model [15] given as follows, $Y_{ijk}=\mu +g_{i}+g_{j}+s_{ij}+r_{ij}+b_{k}+\varepsilon_{ijk}$, where *Y*_*ijk*_ is the observed response value of the cross between parents *i* and *j*; *μ* is the overall mean; *g*_*i*_ is the general combining ability effect of parent *i*; *g*_*j*_ is the general combining ability of the parent *j*; *s*_*ij*_ is the specific combining ability of the cross between parents *i* and *j*; *r*_*ij*_ is the effect of the reciprocal cross between parents *i* and *j*; *b*_*k*_ is the effect of the *k*^*th*^ block; and *ε*_*ijk*_ is the experimental error.

The relative importance of the *gca* and *sca* effects were determined with the general predictive ratio (*gp*r) [20, 21] given as $gpr=(2\sigma_{gca}^{2})/(2\sigma_{gca}^{2}+\sigma_{sca}^{2})$, where $\sigma_{gca}^{2}$ is the variance of the *gca* effect and $\sigma_{sca}^{2}$ is the variance of the *sca* effect. A *gpr* closer to unity indicates the hybrid’s performance is a function of the *gca* alone [20]. The percentage of heterosis was estimated with two methods, the mid-parent heterosis (*mph*) and the better-parent heterosis (*bph*). They are $mph=(F_{1}-mp)*100/mp$ and $bph=(F_{1}-bp)*100/bp$, where *F*_1_ is the mean performance of the *F*_1_ hybrid, $mp=(P_{1}+P_{2})/2$ is the average value of the two parents, *P*_1_ and *P*_2_, and *bp* is the value of the better parent. A test of hypothesis on the significance of the two statistics *mph* and *bph* uses the Student’s t test with respective standard error, $\sqrt{3*MSE/8}$ and $\sqrt{MSE/2}$ where *MSE* is the error variance [22, 23]. The potency ratio (*P*) determined the degree of gene dominance and was computed as $P=(F_{1}-mp)*100/(0.5*(P_{2}-P_{1}))$ [24, 25], with *P*_2_ representing the average performance of the better parent, and *P*_1_ the average performance of the weaker parent. The space of *P* is the set of real numbers. However, *P* = ±1% indicates complete dominance, −1%<*P*<1% means partial dominance, *P* = 0% means absence of dominance and *P*>1% or *P*<−1% is the indication of super-dominance. The positive or negative sign of *P* defines the direction of dominance with respect to the parents [24]. Randomly selected individuals of each parental line were used to create classes of promising parental lines based on their *gca* effects on plant height and fruit- and seed-yield traits. The statistical software R [26], the packages lmdiallel [27] and ape [28] and the online application iTOL [29] were used for data analysis and graphics.

## 3. Results

### 3.1. Mean performances of parents and hybrids

**Fig 1:**
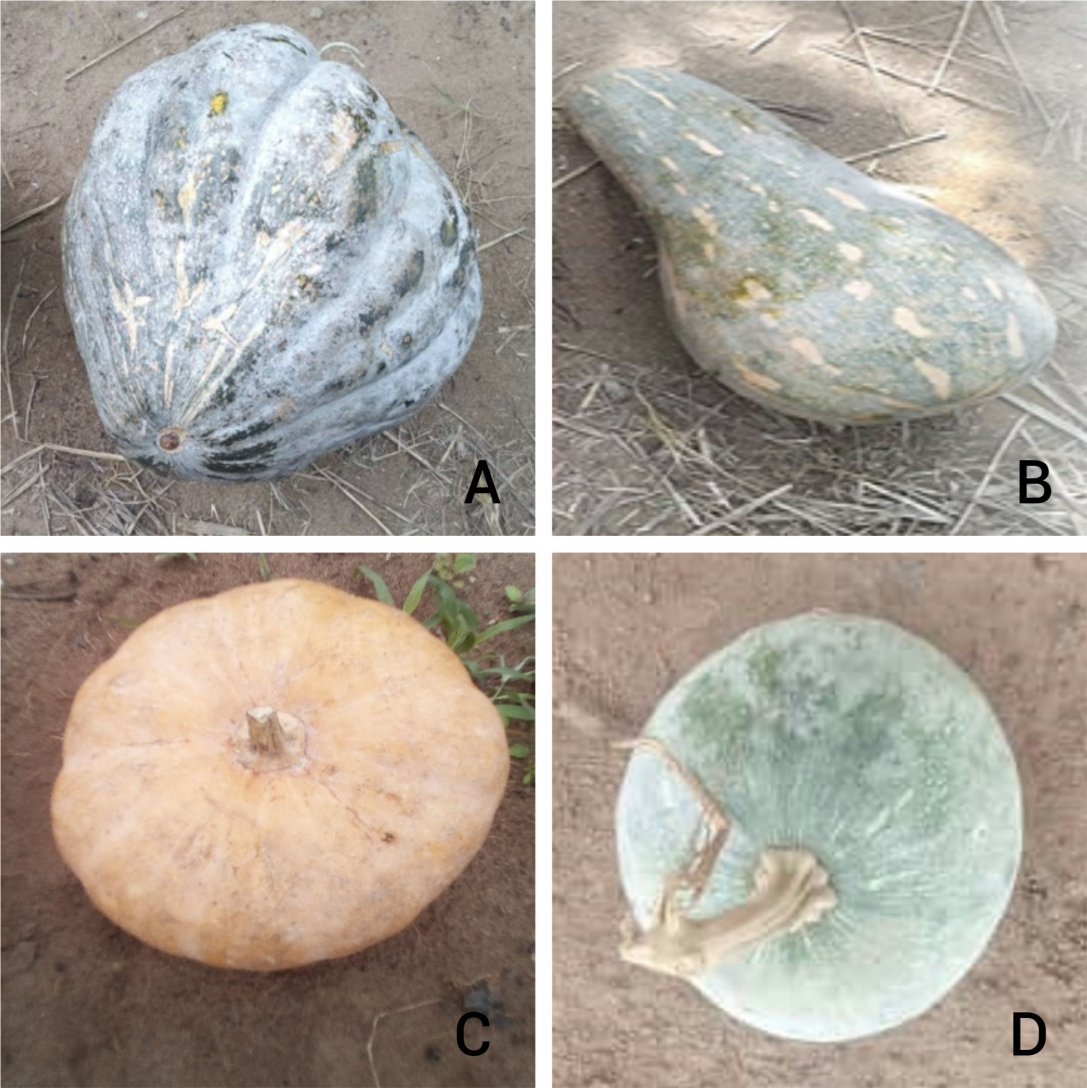
Diversity of the fruits of the parental lines of *Curcubita moschata*. (A) Fruit of the parental line *Soubre*. (B) Fruit of the parental line *Long*. (C) Fruit of the parental line *Oval*. (D) Fruit of the parental line *Zouan-H*.

The means of the plant height, fruit- and seed-yield traits are reported in Table 3. The analysis of variance showed highly significant differences among the parental lines and the hybrid combinations for all the traits studied. Fig 1 shows the diversity of the fruit traits among the parental lines. The method of Tukey for the multiple comparison of means helped to identify significantly different means. It could be seen that the line identified as *Long* has the highest plant height among the four parental lines. The hybrids involving *Long* as a parent have the highest plant height. The *Soubre* parent is characterized by higher number of fruits that have larger dimensions (length, width, and diameter of the fruit) and are the heaviest. Hybrids produced with *Soubre* as female parent have the largest number of fruits, the longest, widest and heaviest fruits. In addition, the fruits of the *Soubre* parents have the heaviest pulp. That trait is also inherited in the hybrids with *Soubre* as a female parent.

**Table 3:** Means of plant height, fruit- and seed-yield traits of the parental lines and the hybrid combinations of *Cucurbita moschata* used in this study.

Variables														
Parental lines			PLH	NFP	LOF	LAF	WOF	DOF	WTM	NOS	WFS	W100	LDS	WIS
Zouan-H			451,64de	1,64cd	23,38c	15,81b	2551,64bc	17,74cd	2001,59cd	334,13c	60,51c	10,03e	1,27c	0,79d
Long			536,75cd	1,40d	25,92bc	8,47f	1011,53d	9,49g	860,56f	300,63c	49,74d	10,23e	1,44c	0,78d
Oval			414,01e	1,32d	18,52e	11,23de	1631,42cd	14,04de	1154,72d	303,32c	58,91cd	11,42de	1,63b	0,82cd
Soubre			462,95de	2,25b	32,77a	19,18a	4589,76a	20,05a	4503,89a	199,49de	82,42b	12,54d	1,56bc	0,88cd
**Hybrids**														
F_1_ (Z_♀_ x L_♂_)		915,00a		1,80cd	25,53bc	9,93ef	2840,00bc	17,90cd	2227,67c	608,30a	57,22cd	11,78d	1,39c	0,92c
F_1_ (L_♀_ x Z_♂_)		912,33a		1,13de	25,27bc	10,03e	2766,00bc	9,90fg	2169,76cd	602,70a	58,82cd	12,09f	1,37c	0,93c
F_1_ (Z_♀_ x O_♂_)		535,67cd		1,79cd	20,93cd	14,97bc	2859,56bc	18,17c	2054,60cd	630,10a	88,49b	15,30c	1,29d	1,10bc
F_1_ (O_♀_ x Z_♂_)		520,33d		1,58cd	20,17cd	14,20bc	2748,54bc	18,00c	2031,67cd	627,80a	87,61b	15,49c	1,33cd	1,18bc
F_1_ (Z_♀_ x S_♂_)		671,67c		2,70b	29,50ab	19,70a	3240,70b	19,47ab	2206,10c	217,00d	91,48ab	20,72b	1,61b	0,97c
F_1_ (S_♀_ x Z_♂_)		678,00c		3,56a	29,63ab	19,70a	4125,33a	18,40bc	3786,63b	431,30b	88,87b	21,33b	1,62b	0,99c
F_1_ (L_♀_ x O_♂_)		939,33a		1,5cd	19,77de	9,63ef	1179,00cd	11,57ef	1002,67e	350,67c	83,59b	11,85d	1,65b	0,89cd
F_1_ (O_♀_ x L_♂_)		952,33a		1,58cd	20.00d	10,26de	1209,67cd	11,87ef	1029,00e	359,67c	58,64cd	12,05d	1,57bc	0,92c
F_1_ (L_♀_ x S_♂_)		855,87ab		2,08c	31,27a	14,30bc	1283,67cd	10,77f	1092,00de	196,33de	101,59a	19,36bc	1,77b	1,33ab
F_1_ (S_♀_ x L_♂_)		876,27ab		3,18ab	31,10a	13,50bcd	1670,33cd	14,23de	1170,99d	310,20c	57,65cd	12,13d	1,76b	1,25ab
F_1_ (O_♀_ x S_♂_)		481,83de		2,16c	26,73b	18,77ab	3782,09b	18,57bc	3711,67b	429,00b	100,23a	26,27a	2,07a	1,49a
F_1_ (S_♀_ x O_♂_)		485,23d		3,16ab	26,13b	18,70ab	4692,67a	20,93a	4634,09a	203,67d	82,42e	25,91a	2,11a	1,46a
Statistics	F	668,076		2,052	25,414	13,043	2615,201	15,694	2248,520	381,520	402,730	15.531	1,590	1,044
	P	<0,001		<0,001	<0,001	<0,001	<0,001	<0,001	<0,001	<0,001	<0,001	<0,001	<0,001	<0,001

Mean values followed by the same letter are not significantly different at the level α = 0.05.

The parental lines *Zouan-H*, *Long* and *Oval* have significantly higher number of seeds per fruit. But the seeds are smaller and lighter. The hybrids produced from these three parents also have the highest number of seeds. In particular, *Z*_♀_ x *L*_♂_, *L*_♀_ x *Z*_♂,_ *Z*_♀_ x *O*_♂_ and *O*_♀_ x *Z*_♂_ have doubled the number of seeds per fruit compared to their parents. Hybrids produced from those three parents have smaller seeds that are lighter. The *Soubre* parent has a smaller number of seeds that are comparatively bigger and heavier. Hybrids involving *Soubre* as a parent have smaller number of seeds per fruit, except *O*_♀_ x *S*_♂_ and *S*_♀_ x *Z*_♂_. But, they have heavier and bigger seeds. In most cases, the hybrids outperformed the parents. The hybrids *S*_♀_ x *O*_♂_ and *O*_♀_ x *S*_♂_ more than doubled their 100-seed weight compared to either parent.

### 3.2. Heterosis and values of the potency ratio for the different traits

#### 3.2.1. Estimates of heterosis in the F1 hybrids

The estimates of heterosis expressed as a percent increase (or decrease) with respect to the average of the two parents (*mph*) and the better parent (*bph*) are presented in Tables 4 and 5, respectively.

**Table 4:** Mid-parent heterosis (in percentage) observed on fruit- and seed-yield traits and plant height of the hybrids.

Mid-parent heterosis (%)												
Hybrids	PLH	NFP	LOF	LAF	WOF	DOF	WTM	NOS	WFS	W100	LDS	WIS
F_1_ (Z_♀_ x L_♂_)	85.22*	20.14ns	3.60ns	-18.2ns	59.18**	31.47**	55.66**	91.57**	3.80ns	16.29*	2.58ns	17.20**
F_1_ (L_♀_ x Z_♂_)	84.68*	-26.67ns	2.53ns	-17.4ns	55.07**	-27.00**	51.61**	89.68**	6.70ns	19.35*	1.11ns	18.47**
F_1_ (Z_♀_ x O_♂_)	23.71ns	20.00ns	-0,11ns	10.72*	36.69*	14.35*	30.15*	97.66**	48.20*	42.66*	-11.03*	36.64**
F_1_ (O_♀_ x Z_♂_)	20.17ns	5.33ns	-3.7ns	5.03ns	31.39*	13.28*	28.74ns	96.72**	46.73*	44.43*	-8.28**	46.58*
F_1_ (Z_♀_ x S_♂_)	46.97ns	42.11ns	5.08ns	12.6*	-9.24ns	3.04ns	-32.18*	-18.67ns	74.78**	83.61**	13.00**	16.17**
F_1_ (S_♀_ x Z_♂_)	48.36ns	87.37**	5.54ns	13.02*	6.04ns	-3.04ns	26.83ns	61.54*	69.79**	89.01**	14.36**	18.56**
F_1_ (L_♀_ x O_♂_)	97.75*	10.29ns	-11.00ns	-2.20ns	89.31**	-1.06ns	-0.49ns	16.13ns	53.87**	9.47ns	7.49*	11.25*
F_1_ (O_♀_ x L_♂_)	99.49**	16.18ns	-9.99ns	1.52ns	-8.46ns	0.89ns	2.12ns	19.11ns	7.94ns	11.32ns	2.28ns	15.00**
F_1_ (L_♀_ x S_♂_)	71.27ns	16.67ns	6.60ns	3.44ns	-54.17**	-27.08**	-59.29**	-21.49ns	99.36**	70.05**	18.00**	60.24**
F_1_ (S_♀_ x L_♂_)	75.20ns	77.78*	6.09ns	-2.44ns	35.02**	26.09**	38.38*	71.56*	22.78*	6.54ns	17.33**	50.60**
F_1_ (O_♀_ x S_♂_)	9.82ns	20.03 ns	4.23ns	23.44*	-46.30**	-19.78*	-58.61**	23.31ns	94.73**	99.29**	29.78**	75.29**
F_1_ (S_♀_ x O_♂_)	10.78ns	77.78*	1.90ns	23.42*	50.86**	23.44*	63.79**	-18.99ns	59.91**	96.28**	32.29**	71.76**

(ns): not significant

(*): significant at *α* = 0.05

(**): significant at *α* = 0.01.

**Table 5:** Better-parent heterosis (in percentage) observed with the agronomic, fruit- and seed-yield traits of the hybrids.

Better-parent heterosis (%)												
Hybrids	PLH	NFP	LOF	LAF	WOF	DOF	WTM	NOS	WFS	W100	LDS	WIS
F_1_ (Z_♀_ x L_♂_)	70.47ns	9.76ns	-1.50ns	-37.20*	11.01ns	0.94ns	11.30ns	81.97**	-5.44ns	15.15*	-3.47ns	16.46**
F_1_ (L_♀_ x Z_♂_)	69.97ns	-32.93ns	-2.50ns	-36.56*	8.06ns	-44.05**	8.40ns	80.17**	-2.79ns	18.18*	-4.86ns	17.72**
F_1_ (Z_♀_ x O_♂_)	18.83ns	9.76ns	-10.5ns	-5.30ns	12.20ns	3.25ns	2.62ns	88.55**	46.24*	33.98*	-20.86**	34.15**
F_1_ (O_♀_ x Z_♂_)	15.21ns	-3.66ns	-13.73ns	-10.20ns	7.69ns	1.47ns	1.50ns	87.65**	44.79*	35.64*	-18.40**	43.90**
F_1_ (Z_♀_ x S_♂_)	45.08ns	20.00ns	-10.00ns	2.71ns	-29.39*	-2.89ns	-51.02**	-35.06ns	51.18**	65.23**	3.02ns	10.23*
F_1_ (S_♀_ x Z_♂_)	46.45ns	58.22ns	-9.58ns	2.71ns	-17.01*	-8.23ns	-8.41ns	28.99ns	46.87*	70.09**	3.40ns	12.50*
F_1_ (L_♀_ x O_♂_)	75.32ns	7.14ns	-23.73ns	-14.20ns	-27.73*	-18.13*	-13.17ns	15.61ns	41.89*	3.77ns	1.23ns	8.54*
F_1_ (O_♀_ x L_♂_)	77.43ns	12.86ns	-22.80	-10.95ns	-26.09*	-15.27ns	-10.89ns	18.58ns	-0.46ns	5.52ns	-3.68ns	12.19*
F_1_ (L_♀_ x S_♂_)	59.29ns	-7.56ns	-4.58ns	-25.40*	-72.10**	-46.28**	-75.75**	-34.39ns	89.24**	54.34**	13.46**	51.14**
F_1_ (S_♀_ x L_♂_)	63.20ns	41.33ns	-5.10ns	-29.61*	-17.59*	-7.28ns	-20.48ns	42.70*	15.90*	-3.27ns	12.82*	42.05**
F_1_ (O_♀_ x S_♂_)	3.90ns	-4.18ns	-18.40ns	-2.10ns	-64.11**	-29.03**	-74.00**	2.20ns	70.14**	89.49**	26.99**	69.32**
F_1_ (S_♀_ x O_♂_)	4.81ns	42.22ns	-20.3ns	-2.55ns	2.24ns	4.25ns	2.89ns	-32.85ns	39.91*	86.62**	29.45**	65.91**

(ns): not significant

(*): significant at *α* = 0.05

(**): significant at *α* = 0.01.

Mid-parent heterosis was significant for all measured traits in the crosses, except the length of the fruit. For the character plant height, significant mid-parent heterosis was observed with hybrids involving the parents *Long* and *Zouan-H*, and the parents *Long* and *Ova*l. Regarding the number of fruits per plant, mid-parent heterosis was significant only in hybrids where *Soubre* is the female parent. None of the estimated better-parent heterosis was significant for the number of fruits per plant. For the length of the fruit, none of the estimated heterosis was significant, whether computed on the basis of the average of the two parents or the better parent. Significant mid-parent heterosis was observed in the hybrids from the crosses between the parental lines *Soubre* and *Oval*, *Soubre* and *Zouan-H*, and in the hybrid *Z*_♀_ x *O*_♂_ for the character width of the fruit. For that character, the significant estimates of better-parent heterosis were negative. They were observed in the hybrids involving the parents *Zouan-H* and *Long*, and *Soubre* and *Long*, whether used as male or female parent. For the diameter of the fruit, significant estimates of mid-parent heterosis were observed in the crosses *Z*_♀_ x *L*_♂_, *S*_♀_ x *L*_♂_, and *S*_♀_ x *O*_♂_. The reciprocal crosses yielded significant negative estimates of mid-parent heterosis. Also, significant estimates of mid-parent heterosis were observed with the hybrids from the cross *Z*_♀_ x *O*_♂_ and its reciprocal. All significant estimates of better-parent heterosis were negative. They were observed in the hybrids *L*_♀_ x *Z*_♂_, *L*_♀_ x *O*_♂_, *L*_♀_ x *S*_♂_, and *O*_♀_ x *S*_♂_. For the weight of the pulp, all estimates of mid-parent heterosis were significant except in the following hybrids *O*_♀_ x *Z*_♂_, *S*_♀_ x *Z*_♂_, *L*_♀_ x *O*_♂_, and *O*_♀_ x *L*_♂_. However, the estimated better-parent heterosis was not significant for the same trait in all hybrids except *Z*_♀_ x *S*_♂_, *L*_♀_ x *S*_♂_, and *O*_♀_ x *S*_♂_. For the character number of seeds per fruit, significant estimates of heterosis computed with either method were seen in the crosses involving *Zouan-H* and *Long* and *Zouan-H* and *Oval* and their reciprocals. Mid-parent heterosis was also significant in the hybrids *S*_♀_ x *Z*_♂_ and *S*_♀_ x *L*_♂_ and better-parent heterosis was significant in the latter hybrid. With the weight of fresh seeds per fruit, heterosis whether estimated with the average of the two parents or with the better parent, was significant in all crosses except *Z*_♀_ x *L*_♂_, *L*_♀_ x *Z*_♂_ and *O*_♀_ x *L*_♂_. And significant heterosis was found for the 100-seed weight in all hybrids except *L*_♀_ x *O*_♂_, *O*_♀_ x *L*_♂_ and *S*_♀_ x *L*_♂_. That observation applied to the two methods of estimation. Regarding the character length of the dry seed, mid-parent heterosis was significant in all hybrids except those from crosses involving *Zouan-H* and *Long*, and in the hybrid *O*_♀_ x *L*_♂._ For the same character, estimate of better-parent heterosis was significant only in crosses where the parents were *Zouan-H* and *Oval*, *Long* and *Soubre*, and *Oval* and *Soubre*. In hybrids from the crosses involving *Zouan-H* and *Oval*, better-parent heterosis was negative. For the width of the dry seed, heterosis computed with either method was significant in all hybrids. These results implied that the different hybrids exhibited varied performances according to the traits observed.

#### 3.2.2. Effects of gene dominance

The effects of gene dominance are determined by the values of the potency ratio. They are reported in Table 6. In the absence of a significance test, values were interpreted as they were. We did not assume a value -0.99 to equate -1.00 or a value 0.02 to equate 0.00, without statistical support. Doing so would give totally different interpretations of gene expression in many cases of the quantitative analysis.

**Table 6:** Potency ratio for the fruit- and seed-yield traits and plant height of the hybrids of *Cucurbita moschata*.

Potency ratio												
Hybrids	PLH	NFP	LOF	LAF	WOF	DOF	WTM	NOS	WFS	W100	LDS	WIS
F_1_ (Z_♀_ x L_♂_)	9.89	2.50	0.69	-0.60	1.37	1.04	1.40	17.35	0.39	16.50	2.43	27.00
F_1_ (L_♀_ x Z_♂_)	9.83	-0.33	0.49	-0.57	1.28	-0.90	2.59	16.99	0.69	9.80	0.18	29.00
F_1_ (Z_♀_ x O_♂_)	5.47	1.81	-0.10	0.63	1.67	1.23	1.12	10.10	35.98	6.58	0.89	19.67
F_1_ (O_♀_ x Z_♂_)	4.64	0.08	-0.32	0.30	1.43	1.14	1.07	20.01	34.88	6.86	-0.30	25.00
F_1_ (Z_♀_ x S_♂_)	37.96	2.29	0.30	1.30	-0.32	0.50	-0.84	-0.74	4.79	7.52	1.10	3.00
F_1_ (S_♀_ x Z_♂_)	39.61	5.30	0.33	1.31	0.21	-0.43	0.70	2.44	4.47	8.00	1.58	3.44
F_1_ (L_♀_ x O_♂_)	7.56	3.51	-0.66	-0.20	0.46	0.09	-0.03	36.20	6.38	1.72	1.21	4.50
F_1_ (O_♀_ x L_♂_)	7.78	3.26	-0.60	0.10	-0.36	0.05	0.15	42.90	0.94	2.06	0.37	6.00
F_1_ (L_♀_ x S_♂_)	8.90	0.66	0.56	0.10	-0.85	-0.76	-0.87	-0.98	51.85	6.90	4.50	10.00
F_1_ (S_♀_ x L_♂_)	10.19	3.19	0.51	-0.11	0.55	0.72	0.57	3.54	3.84	0.65	4.33	8.40
F_1_ (O_♀_ x S_♂_)	1.76	0.77	0.15	0.90	-0.97	-0.94	-0.99	1.13	6.61	25.52	13.57	2.00
F_1_ (S_♀_ x O_♂_)	1.93	2.96	0.07	0.88	1.07	1.29	1.08	-0.92	4.19	24.88	14.71	20.33

The gene(s) controlling plant height exhibited a super-dominance in all the hybrids examined because the potency ratio was greater than one (*P* > 1) in all the crosses of the inbred lines of *Cucurbita moschata* used in this study. For the number of fruits per plant, a partial dominance was observed in the following F1 hybrids *L*_♀_ x *Z*_♂_, *O*_♀_ x *Z*_♂_, *L*_♀_ x *S*_♂_, and *O*_♀_ x *S*_♂_ because -1 < *P* < 1 and *P* ≠ 0, and a super-dominance in the expression of the character in all the other hybrids. The negative value of the potency ratio observed in the F1 hybrid *L*_♀_ x *Z*_♂_ indicates a reduction in the number of fruits per plant for that hybrid. Partial dominance was observed in the expression of the length of the fruits in all hybrids. For F_1_ (*Z*_♀_ x *O*_♂_) and F_1_ (*L*_♀_ x *O*_♂_) and their respective reciprocals, the values of the potency ratio were negative, indicating a relative reduction of the length of the fruits in those hybrids. The F1 hybrids issued from the cross *Z*_♀_ x *S*_♂_ and its reciprocal showed a super-dominance of the gene(s) determining the width of the fruits in those hybrids. In all the other hybrid combinations, partial dominance was observed in the expression of the width of the fruit, and the values of the potency ratio were negative for the following F1 hybrids *Z*_♀_ x *L*_♂_, *L*_♀_ x *Z*_♂_, *L*_♀_ x *O*_♂_, and *S*_♀_ x *L*_♂_. For the weight of the fruit, a super-dominance in the expression of the trait was observed in the F1 hybrids *Z*_♀_ x *L*_♂_, *L*_♀_ x *Z*_♂_, *Z*_♀_ x *O*_♂_, *O*_♀_ x *Z*_♂_, and *S*_♀_ x *O*_♂_. All the other hybrids exhibited partial dominance, and the values of the potency ratio were negative for the following F1 hybrids *Z*_♀_ x *S*_♂_, *O*_♀_ x *L*_♂_, *L*_♀_ x *S*_♂_, and *O*_♀_ x *S*_♂_. Expression of the diameter of the fruit varied too, according to the hybrids. A super-dominance in the expression of the diameter of the fruit was observed in the F_1_ (*Z*_♀_ x *L*_♂_), F_1_ (*Z*_♀_ x *O*_♂_), F_1_ (*O*_♀_ x *Z*_♂_), and F_1_ (*S*_♀_ x *O*_♂_), and partial dominance for the character in all the other F1 hybrids. Four F1 hybrids had a negative value of the potency ratio for the diameter of the fruit. For the weight of the pulp, the expression of the trait varied according to the hybrids, but in a different pattern. In the F1 hybrids from the crosses involving *Zouan-H* and *Long*, *Zouan-H* and *Oval*, their respective reciprocals and the F_1_ (*S*_♀_ x *O*_♂_), the gene(s) that determined the weight of the pulp were super-dominant. In all the other hybrids, partial dominance was observed in the expression of the character with some cases of negative value of the potency ratio. The expressions of the examined seed-related traits were mostly super-dominant. For the number of seeds per fruit, super-dominance was observed in the determination of the character in all F1 hybrids except F_1_ (*Z*_♀_ x *S*_♂_), F_1_ (*L*_♀_ x *S*_♂_), and F_1_ (*S*_♀_ x *O*_♂_) where -1 < *P* < 0. The gene(s) governing the weight of fresh seeds were super-dominant in all F1 hybrids except F_1_ (*Z*_♀_ x *L*_♂_), F_1_ (*L*_♀_ x *Z*_♂_), and F_1_ (*O*_♀_ x *L*_♂_) where partial dominance was observed. A super-dominance was observed in all hybrids for the 100-seed weight except F_1_ (*S*_♀_ x *L*_♂_) where we observed partial dominance. For the length of the dry seed, partial dominance was observed in the F_1_ (*L*_♀_ x *Z*_♂_), F_1_ (*Z*_♀_ x *O*_♂_), F_1_ (*O*_♀_ x *Z*_♂_), and F_1_ (*O*_♀_ x *L*_♂_). In all the other hybrid combinations, the expression of the length of the dry seed was characterized by a super-dominance of the long seed. And the width of the seed was characterized by a super-dominance of the wide seed in all hybrids studied.

#### 3.2.3. Analysis of variance of the *gca* and *sca* of the traits

The examined traits significantly differed according to blocks (Table 7). Across all the blocks, we observed highly significant *gca* effects of the female parents for all traits studied, indicating a very large variation of the *gca* effects of the inbred lines when used as females. When used as male parents, the *gca* effects were significant for all the traits except plant height, length of the fruit, width of the fruit and length of the seed. For all the traits, the variation in *gca* of the female parent was far greater than that of the male parent. Highly significant *sca* effects were observed for all the traits studied. The reciprocal effects were highly significant for weight and diameter of the fruit, weight of the pulp, number of seeds per fruit, weight of the fresh seeds and 100-seed weight, indicating strong maternal effects on those traits. The general predictive ratio is given as $gpr=2\sigma_{gca}^{2}/(2\sigma_{gca}^{2}+\sigma_{sca}^{2})$. A value of *gpr* = 2/3 indicates equal contribution of additive and non-additive gene effects in the determination of a trait. A value of *gpr* < 2/3 means the predominance of non-additive gene effects over the additive ones in the expression of the trait. And a value of *gpr* > 2/3 means that additive gene effects predominantly determine the trait. Especially, a *gpr* closer to 1 means only additive gene effects control the expression of the trait. In this study, the general predictive ratio was greater than two-third for all the traits except weight of the fresh seed and width of the dry seed. We may infer from those observations that most of the characters under this study are predominantly determined by the effects of additive gene(s). But, weight of the fresh seed and width of the dry seed are mostly controlled by non-additive gene(s). In particular, traits related to the fruit have a general predictive ratio greater than 0.9 and very close to 1, meaning that they are almost solely determined by additive gene effects.

**Table 7:** Analysis of variance of the *gca* and *sca* effects based on the linear diallel model, and general predictive ratio of the fruit-, seed-yield traits and plant height of four inbred lines and their hybrid combinations of *Cucurbita moschata*.

		Mean Square											
SOV	df	PLH	NFP	LOF	LAF	WOF	DOF	WTM	NOS	WFS	W100	LDS	WIS
Block	2	3961432**	6.198**	2854.2**	933.7**	261077454**	2597.1**	223039259**	363343**	24797**	243.2**	0.133*	0.076ns
GCA(female)	3	8706682**	153.516**	10970.5**	8056.6**	431617966**	4777.5**	468478919**	5827578**	29220**	6228.8**	20.709**	7.501**
GCA(male)	3	11451ns	60.834**	5.8ns	7.5ns	239998329**	2560.2**	397639672**	1045979**	41302**	469.6**	0.008ns	0.178**
SCA	6	7014551**	38.308**	248.3**	389.8**	34229753**	109.7**	19207823**	2821242**	57108**	4376.9**	6.326**	10.520**
Reciprocal	3	5135ns	1.527ns	13.1ns	16.0ns	42467338**	517.0**	17294741**	962334**	10541**	482.9**	0.045ns	0.066ns
Residuals	1710	98411	1.389	57.5	11.8	2131174	18.7	2110005	27171	664	18.0	0.039	0.031
*gpr*		0.713	0.889	0.989	0.976	0.962	0.989	0.979	0.918	0.506	0.740	0.867	0.588

The estimates of general combining ability varied across parental lines as well as the traits, with negative and positive values (Table 8). In general, negative *gca* effects would be desirable for plant height. We would want the plant to complete its vegetative growth early enough in order to allocate larger proportion of dry matter to the development of the fruits and the seeds. Positive *gca* effects would be preferred for fruit- and seed-related traits because they are the commonly harvestable organs that determine yield. The parental line *Soubre* had negative *gca* effects for plant height and positive *gca* effects for all the fruit- and seed-related traits except the number of seeds per fruit. The parent *Soubre* would be suitable for developing a shorter *C*. *moschata* hybrid with higher number of large fruits, and large seeds. The parent *Zouan-H* has negative *gca* effects for plant height, and positive *gca* effects for fruit- and seed-related traits except the number of fruits per plant and fruit length. Both *Soubre* and *Zouan-H* have very high estimates of *gca* effects on the weight of the fruit and pulp mass. If interest lies in a longer vegetative growth with reduced fruit- and seed-yield, the inbred line *Long* would be preferred as it has very high *gca* effects for plant height and negative or non-significant *gca* effects for fruit- and seed-related traits, except number of seeds per fruit. The parental line *Oval* would be suitable for reducing plant height and increasing the number of seeds per fruit. It has significant negative *gca* effects for plant height, and positive *gca* effects for number of seeds per fruit. Except the conspicuous difference in plant height, *Long* and *Oval* have many similarities, especially their negative *gca* effects on the fruit and seed traits.

**Table 8:** Estimation of the general combining ability (*gca*) of the parental lines and the specific combining ability (*sca*) of the F1 hybrids for the fruit- and seed-yield traits and plant height of *Cucurbita moschata* used in this study.

		PLH	NFP	LOF	LAF	WOF	DOF	WTM	NOS	WFS	W100	LDS	WIS
Parental lines		gca of parents											
Zouan-H		-25.93**	-0.07*	-0.69**	0.76**	302.82**	1.44**	99.21ns	91.54**	1.07ns	-0.88**	-0.19*	-0.19*
Long		147.48**	-0.29**	0.18ns	-3.72**	-729.70**	-3.17**	-633.79**	21.99**	-8.49*	-3.08**	-0.04ns	-0.04ns
Oval		-75.21**	-0.25**	-4.06**	-0.67****	-412.43**	-0.36*	-464.96**	16.58**	4.23*	-0.66**	0.06ns	0.06ns
Soubre		-46.34**	0.62**	4.57**	3.62**	839.31**	2.09**	999.54**	-108.12**	3.20*	3.30**	0.16*	0.17*
(SE)		(39)	(0.05)	(0.37)	(0.18)	(124.15)	(0.36)	(235.99)	(6.94)	(2.39)	(0.44)	(0.01)	(0.009)
Mating		sca of crosses											
Z ⊗ L		124.16**	-0.22*	1.49**	-1.32**	614.75**	-0.09ns	480.23**	120.09**	-7.67*	0.36*	0.03*	0.03*
Z ⊗ O		-38.83*	-0.04ns	-0.10ns	0.23ns	297.98**	1.28**	155.57ns	151.01**	9.63**	0.06ns	-0.15*	-0.15*
Z ⊗ S		79.14**	0.54**	0.27ns	1.06**	243.60**	-0.32ns	-186.10ns	-40.79*	12.79**	3.06**	-0.05*	-0.05*
L ⊗ O		205.60**	0.34**	-1.64**	-0.06ns	-278.65**	-1.47**	-138.43ns	-42.78*	2.26ns	-1.18*	-0.04*	-0.04*
L ⊗ S		96.40**	0.26**	1.01**	-0.20ns	391.90**	0.02ns	-276.93**	27.42*	11.79**	-0.02ns	-0.05*	-0.05*
O ⊗ S		-63.24**	0.24**	0.51	1.52**	339.50**	0.13ns	151.24ns	-21.00*	10.77**	6.57**	0.27*	0.27*
(SE)		(35.85)	(0.11)	(0.69)	(0.33)	(226.67)	(0.67)	(235.99)	(12.67)	(4.38)	(0.82)	(0.02)	(0.02)

(ns) means not significant

(*) means significant at *α* = 0.05

(**) means significant at *α* = 0.01; (⊗) stands for direct cross and reciprocal; (SE) means standard error of the gca or sca effects.

Based on the *sca* effects, we may affirm that crosses involving the parental line *Long* result in hybrids with increased plant height. Crosses between *Zouan-H* and *Long* increase fruit weight, pulp weight, and number of seeds per fruit, along with an increased plant height. And crosses between *Zouan-H* and *Oval* produce hybrids with significantly reduced plant height, increased fruit weight and increased number of seeds per fruit. Combination of crosses involving the parent *Soubre* results in hybrids with increased fruit yield. And hybrids from crosses involving *Oval* and *Soubre* have reduced plant height, increased fruit weight and increased seed weight.

**Fig 2:**
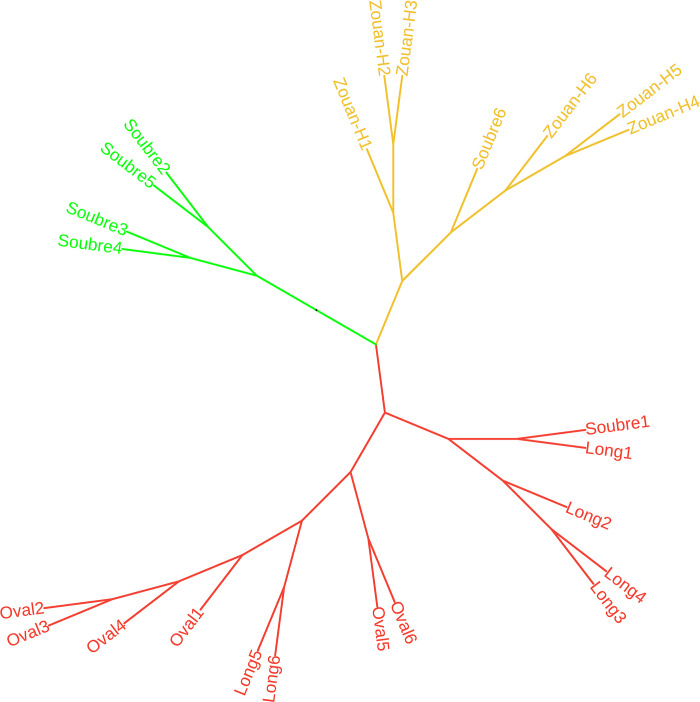
Classification tree of the parental lines, based on their general combining ability effects.

Random samples from each inbred line in each of the three blocks were used to develop a classification model, using the *gca* effects of the parental lines. Based on the within-group sum-of-squares, a three-group model was found appropriate because it had the minimum error variance. Fig 2 is the classification tree that assembled the parental lines in three groups based on their *gca* effects, each group with a distinctive color. The first group is identified with the green color and it assembled the parental line *Soubre*. The *Soubre* line used as a parent has a very high general combining ability effect on fruit traits such as weight of the fruit, diameter of the fruit, length of the fruit, weight of the pulp, and number of fruits per plant. It also has a high positive *gca* effect on the 100-seed weight. The second group is identified with the orange color and includes the parental line *Zouan-H*. It is characterized by a significantly high *gca* effect on the weight of the fruit, the diameter of the fruit, and the number of seeds per fruit but the seeds have a reduced size. The third group, identified with the red color, includes the parental lines *Long* and *Oval*. They both have significant positive *gca* effects on the number of seeds per fruit, and significant negative *gca* effects on all the fruits traits and many of the seed traits. The parental line *Long* is mostly characterized by an extended vegetative growth. Hence, *Soubre* would be a promising line in a breeding program where improvement of fruit yield is the objective. The parent *Long* would be promising in a program to develop hybrids for extended vegetative development. For improvement of the seed traits, the parental lines *Oval*, *Long* and *Zouan-H* could serve as a female parent if an increase in the number of seeds per fruit is the objective. The parent *Soubre* could also serve as a female parent if the objective is to increase the size of the seeds.

## 4. Discussion

The evaluation of the parental lines with respect to the fruits, the seed traits and plant height showed that the lines *Soubre*, *Oval*, *Long*, and *Zouan-H* have very contrasting characters, and pointed out the existence of a large genetic diversity in the *Cucurbita moschata* germplasm. In fact, the genetic diversity of this species is considerable in terms of the shapes, the forms and the sizes of the fruits and the seeds and their growth cycle [30]. The phenotypic variability in these parental lines has already been described [31]. The phenotypic variability observed in the four lines can be used in selection program for the genetic improvement of the traits of interest in *Cucurbita moschata* [32, 33]. The study of the performances of the parents and their F1 hybrid combinations revealed significant differences between the genotypes for each of the characters evaluated in this work. Some F1 hybrids obtained much better performances than others for each trait studied. The hybrids could be proposed as candidates in breeding programs aimed at improving traits where theses hybrids have recorded their best performances [34].

Significant positive heterosis effects were observed in different hybrids for several traits. This hybrid performance could be a consequence of the large genetic distance separating the different parental lines from each other [35]. Hybrids resulting from genetically distant parents express a heterosis effect [36]. And hybrid vigor is based on the complementation of the gametic contributions of the parents by favorable dominant genes [37] and therefore, the expression of the heterosis effect could be linked in part to the non-additive action of genes, dominance and epistasis. The detection of heterosis in a species is an important step for the improvement of the species. Indeed, the expression of heterosis by hybrids for a given trait indicates their potential to produce superior cultivars through the selection of transgressive segregants in segregating populations [38, 39]. Heterosis breeding is a potential tool to improve the quantity, quality and productivity of bitter gourd [40], which cannot be done by traditional methods.

Analysis of variance of the *gca* and *sca* revealed significant general and specific combining ability effects for the evaluated traits. These results suggested the involvement of additive and non-additive gene actions in the expression of the characters. Estimates of the general predictive ratio, $gpr=2\sigma_{gca}^{2}/(2\sigma_{gca}^{2}+\sigma_{sca}^{2})$, for each trait varied between 0.506 and 0.989 with most estimates being greater than 0.900. A *gpr* estimate of 2/3 is the indication of equal variance of the *gca* and the *sca* in the distribution of the variances among the components. For the traits studied in this experiment, the *gpr* estimates were greater than 2/3 for all traits except the weight of the fresh seeds and the width of the dry seed. This indicated the preponderance of additive gene effects in the genetic control of most of the traits studied. The average performance of a genotype in a series of hybrid combinations will be most relevant in the design of a breeding procedure in cultivar development. If a particular cultivar has a high *gca* for a trait, it means that the cultivar would be a valuable parent in the breeding program to improve that trait [41]. In our study, *Soubre* is the best general combiner for a large number of characters studied with the exception of the number of seeds per fruit and plant height. The lines *Oval*, *Long* and *Zouan-H* stand out as the best overall combiners for number of seeds per fruit. *Long* is the best general combiner for plant height. Thus, each of the four parental lines could be used in a selection program to improve the traits of interest in *Cucurbita moschata*. Also, the significant *gca* values ​​for a given trait revealed that the selection and hybridization methods would lead to interesting genetic improvement for the trait, due to the accumulation of desirable and favorable alleles from both parents in the targeted genotype [34].

The best combination in terms of *gca* effects consistently involved one or two best overall combiners as parents. In other words, most crosses resulting in high *gca* involved at least one parent with a high *gca* (high × high, high × low) for the trait in question. The current results are in line with those reported elsewhere [42] based on watermelon crosses with high *gca* effects from parents with either high × high or high × low *gca* effects. The crossover having a high significant value of *sca* involving two best general combiners, would be due to the additive gene action and the additive × non-additive gene interaction which are fixable [36, 43]. For a given character, the best combination in terms of *gca* obtained with a cross involving a single parent having a high *gca* is the result of the additive gene interaction × dominance in the expression of the trait [44] and therefore, the diversity of values of parental *gca* would play an important role in the production of hybrids with significant *gca* values [36, 45]. However, for a given trait, parents with poor general combining skills sometimes give good combinations when crossed with each other, as is the case here in the cross between *Zouan-H × Long* for the weight of the fruit and the weight of the pulp. Significant *sca* effects resulting from crosses of parents with low *gca* indicate the presence of epistasis (non-allelic interaction) at heterozygous loci that are not fixable [44]. It is therefore suggested to use these crosses for the selection of a plant in later generations of breeding [44]. It should also be noted that non-significant *sca* can be obtained after a crossover involving two best general combiners [14]. Therefore, it cannot be generalized that parents with high *gca* effects could only produce good hybrids [46]. Furthermore, combinations having recorded high *sca* values can be improved through conventional genetic improvement methods such as biparental crosses and selective diallel crosses, subsequently, followed by the pedigree selection method, to break any epistatic link that may occur, in order to isolate transgressive segregants [45, 47, 48].

## 5. Conclusion

Our study helped to test four parental lines of *Cucurbita moschata* and their twelve F1 hybrids from a complete diallel cross for the evaluation of fruit and seed traits and plant height. It appears from our work that there is very high genetic diversity among the parental lines *Soubre*, *Long*, *Oval*, and *Zouan-H* and their descendants. Heterosis effects relative to the average of the two parents were observed in the hybrids for all traits except fruit length. With respect to the better parent, heterosis for different combinations of parents was significant for all traits except plant height, number of fruit per plant and fruit length. Significant *gca* effects were observed for all traits for the different parents. The traits studied were predominantly under the influence of genes with additive effects. The *Soubre* parental line proved to be the best combiner for several traits. However, *Zouan-H* was the best combiner for the number of seeds while *Long* was the best combiner for longer vegetative development. *Oval* was the best candidate for number of seeds per plant and for shorter plants. Significant *sca* effects were recorded at some crosses for each trait, suggesting that these crosses can be used for the development of new and more productive hybrids.

## Supporting information

S1 Data (CSV)

S2 Data (CSV)
